# Effectiveness of Face Masks in Preventing Airborne Transmission of SARS-CoV-2

**DOI:** 10.1128/mSphere.00637-20

**Published:** 2020-10-21

**Authors:** Hiroshi Ueki, Yuri Furusawa, Kiyoko Iwatsuki-Horimoto, Masaki Imai, Hiroki Kabata, Hidekazu Nishimura, Yoshihiro Kawaoka

**Affiliations:** a Division of Virology, Department of Microbiology and Immunology, Institute of Medical Science, University of Tokyo, Tokyo, Japan; b Division of Pulmonary Medicine, Department of Medicine, Keio University School of Medicine, Tokyo, Japan; c Clinical Research Division, Virus Research Center, National Hospital Organization Sendai Medical Center, Sendai, Japan; d Department of Special Pathogens, International Research Center for Infectious Diseases, Institute of Medical Science, University of Tokyo, Tokyo, Japan; e Department of Pathobiological Sciences, School of Veterinary Medicine, University of Wisconsin–Madison, Madison, Wisconsin, USA; University of Michigan–Ann Arbor

**Keywords:** COVID-19, N95 masks, SARS-CoV-2, aerosols, droplets, face masks

## Abstract

Guidelines from the CDC and the WHO recommend the wearing of face masks to prevent the spread of coronavirus (CoV) disease 2019 (COVID-19); however, the protective efficiency of such masks against airborne transmission of infectious severe acute respiratory syndrome CoV-2 (SARS-CoV-2) droplets/aerosols is unknown. Here, we developed an airborne transmission simulator of infectious SARS-CoV-2-containing droplets/aerosols produced by human respiration and coughs and assessed the transmissibility of the infectious droplets/aerosols and the ability of various types of face masks to block the transmission. We found that cotton masks, surgical masks, and N95 masks all have a protective effect with respect to the transmission of infective droplets/aerosols of SARS-CoV-2 and that the protective efficiency was higher when masks were worn by a virus spreader. Importantly, medical masks (surgical masks and even N95 masks) were not able to completely block the transmission of virus droplets/aerosols even when completely sealed. Our data will help medical workers understand the proper use and performance of masks and determine whether they need additional equipment to protect themselves from infected patients.

**IMPORTANCE** Airborne simulation experiments showed that cotton masks, surgical masks, and N95 masks provide some protection from the transmission of infective SARS-CoV-2 droplets/aerosols; however, medical masks (surgical masks and even N95 masks) could not completely block the transmission of virus droplets/aerosols even when sealed.

## Abstract (precis)

Airborne simulation experiments showed that cotton masks, surgical masks, and N95 masks provide some protection from the transmission of infective SARS-CoV-2 droplets/aerosols; however, medical masks (surgical masks and even N95 masks) could not completely block the transmission of virus droplets/aerosols even when sealed.

## OBSERVATION

The potential for severe acute respiratory syndrome coronavirus 2 (SARS-CoV-2) transmission via infective droplets and aerosols (1), coupled with guidelines from the CDC (https://www.cdc.gov/coronavirus/2019-ncov/prevent-getting-sick/diy-cloth-face-coverings.html) and WHO (https://www.who.int/emergencies/diseases/novel-coronavirus-2019/advice-for-public/when-and-how-to-use-masks) recommending the wearing of face masks to prevent the spread of CoV disease 2019 (COVID-19), prompted us to evaluate the protective efficiency of face masks against airborne transmission of infectious SARS-CoV-2 droplets/aerosols.

**FIG 1:**
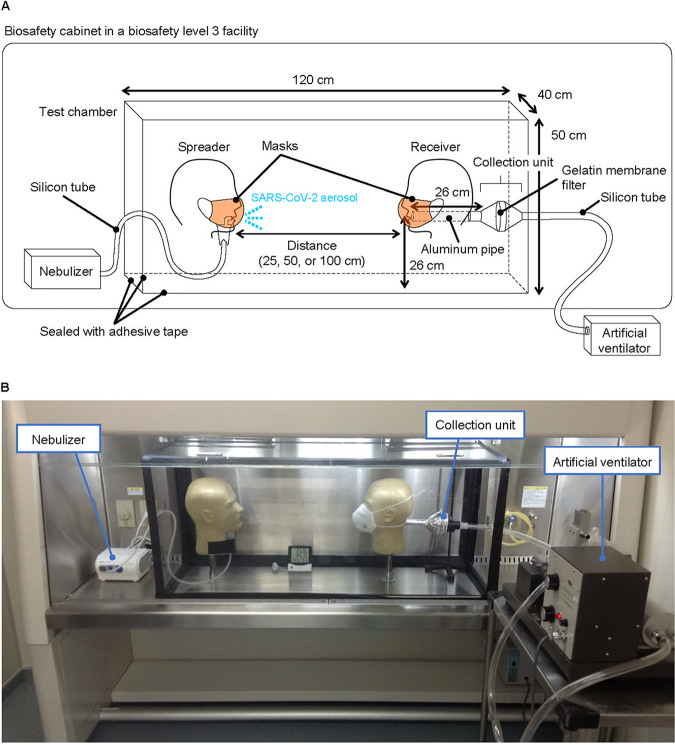
Simulation system for airborne transmission of virus droplets/aerosols. Schematic image (A) and a photograph (B) of the system. A test chamber for airborne transmission experiments was constructed in a BSL3 facility, and two mannequin heads were placed facing each other. One mannequin head was connected to a customized compressor nebulizer and exhaled a mist of virus suspension through its mouth to mimic a viral spreader. The other mannequin head was connected to an artificial ventilator through a virus particle collection unit. Tidal breathing, conducted by the artificial ventilator, was set to a lung ventilation rate representative of a steady state in adults (i.e., 0.5 liter of tidal volume, a respiratory rate of 18 breaths/min, and a 50% gas exchange rate). Face masks were attached to the mannequin heads according to each manufacturer’s instructions.

**FIG 2:**
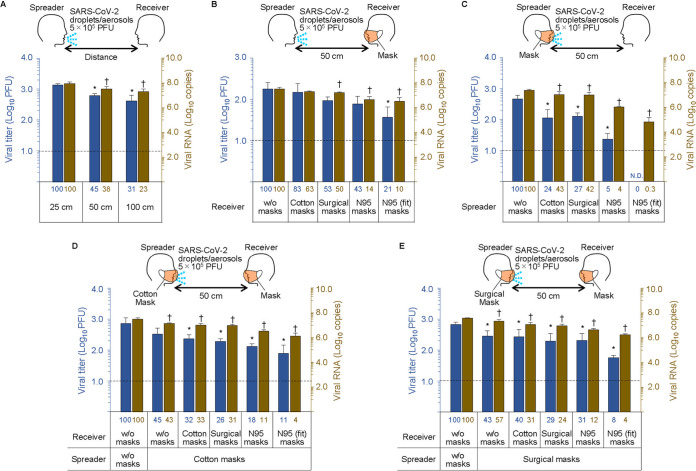

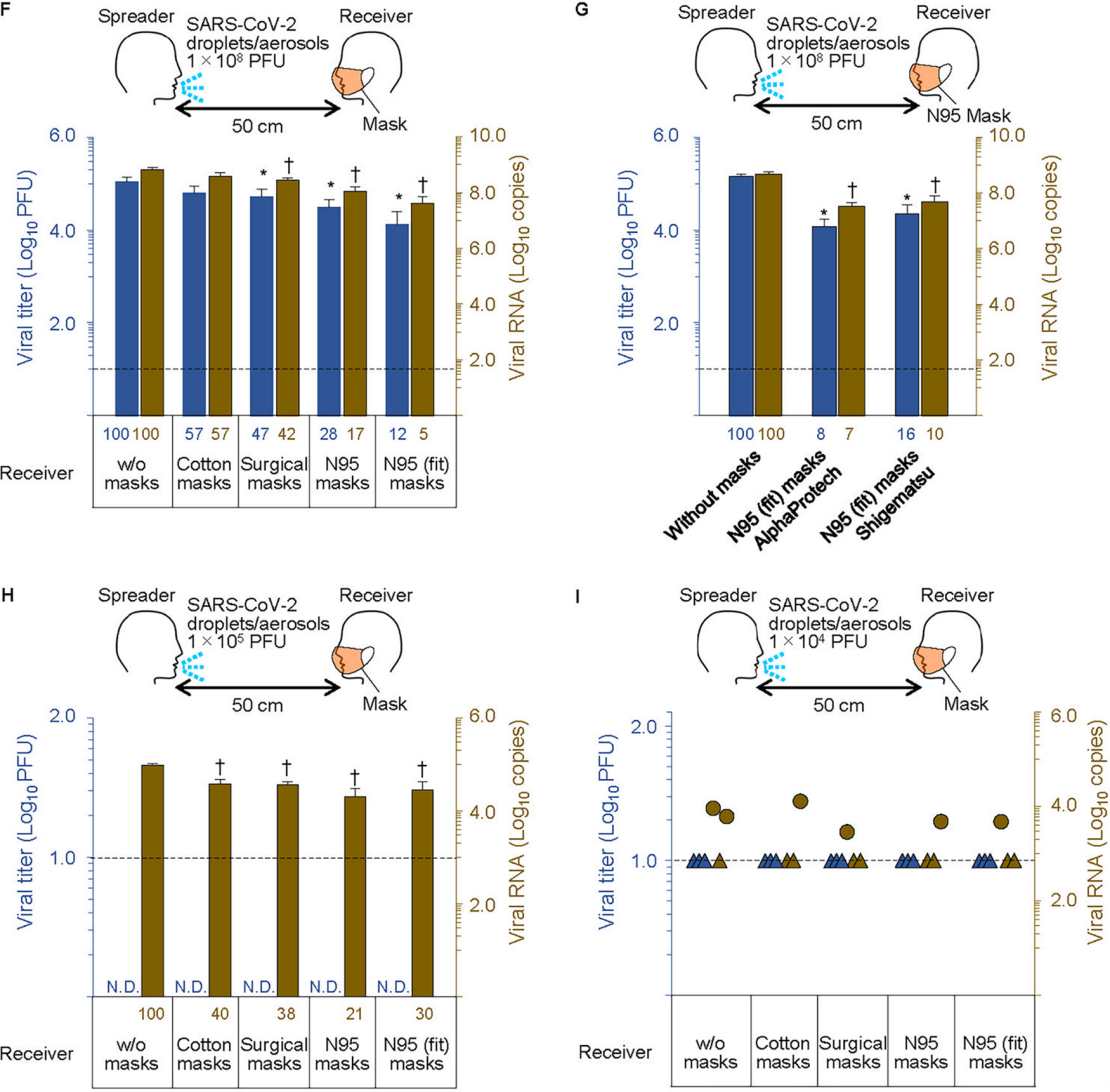
Mask protective efficiency against SARS-CoV-2 droplets/aerosols. The nebulizer was charged with virus suspension (5 × 10^5^ PFU [A to E], 1 × 10^8^ PFU [F and G], 1 × 10^5^ PFU [H], and 1 × 10^4^ PFU [I]) to generate droplets/aerosols and exhaled continuously to simulate a mild cough at a flow speed of 2 m/s for 20 min. Face masks were attached to the mannequin heads, and the viral loads and infective virus that passed through the masks were measured by use of a plaque assay and quantitative real-time reverse transcription PCR (qRT-PCR), respectively. The N95 masks were evaluated using the following two conditions: the mask fit naturally along the contours of the mannequin’s head, or the edges of the N95 masks were sealed with adhesive tape. The blue bars and dots and the *y* axis on the left show virus titers. The brown bars and dots and the *y* axis on the right show the copy numbers of viral RNA. The numbers below the bars show the percentages relative to the leftmost control bar values. Triangles in panel I indicate that the value was below the detection limit. Data are presented as means ± standard deviations (SD). ND, none detected; w/o, without. The experiments were repeated three times (*n* = 3). * and † indicate significant differences from values for the control group (the leftmost column) (*P < *0.05).

We developed an airborne transmission simulator of infectious droplets/aerosols produced by human respiration and coughs and assessed the transmissibility of the infectious droplets/aerosols produced and the ability of various types of face masks to block the transmission (Fig. 1, and see the Methods section in Text S1 in the supplemental material for additional details). A test chamber for airborne transmission experiments was constructed in a biosafety level 3 (BSL3) facility, and two mannequin heads were placed facing each other. One mannequin head was connected to a customized compressor nebulizer and exhaled a mist of virus suspension through its mouth, mimicking a virus spreader. The nebulizer was charged with 6 ml of virus suspension at the viral doses in culture medium indicated in Fig. 2 (without fetal calf serum) or diluted in phosphate-buffered saline to generate droplets/aerosols, and the respiration was exhaled continuously, simulating a mild cough at a flow speed of 2 m/s (2) for 20 min. Although the initial particle size exhaled was 5.5 ± 0.2 μm in mass median diameter (particle size percentages were as follows: <3 μm, 20%; 3 to 5 μm, 40%; >5 to 8 μm, 40% [3]), some of the droplets likely gradually evaporated and changed to aerosols. Therefore, both droplets and aerosols were likely present in the chamber. The other mannequin head was connected to an artificial ventilator through a virus particle collection unit. Tidal breathing, conducted by the artificial ventilator, was set to a lung ventilation rate representative of a steady state in adults. Face masks were attached to the mannequin heads, and the viral loads and infective virus that passed through the masks were measured by use of a plaque assay and quantitative real-time reverse transcription PCR (qRT-PCR), respectively.

10.1128/mSphere.00637-20.1 TEXT S1 Supplemental material, methods, and references. Download Text S1, DOCX file, 0.05 MB. Copyright © 2020 Ueki et al. 2020 Ueki et al. This content is distributed under the terms of the Creative Commons Attribution 4.0 International license.

Viral loads in the inhalation droplets/aerosols were inversely proportional to the distance between the virus spreader and the virus receiver; however, infectious virus was detected even 1 m away (Fig. 2A). The blue bars and the brown bars in the figures show the viral titers and viral RNA copy numbers, respectively. The numbers below each bar show the percentages relative to the leftmost control column values. When a mannequin exposed to the virus was equipped with various masks (cotton mask, surgical mask, or N95 mask), the uptake of the virus droplets/aerosols was reduced. A cotton mask led to an approximately 20% to 40% reduction in virus uptake compared to no mask (Fig. 2B). The N95 mask had the highest protective efficacy (approximately 80% to 90% reduction) of the various masks examined; however, infectious virus penetration was measurable even when the N95 mask was completely fitted to the face with adhesive tape (Fig. 2B). In contrast, when a mask was attached to the mannequin that released virus, cotton and surgical masks blocked more than 50% of the virus transmission, whereas the N95 mask showed considerable protective efficacy (Fig. 2C). There was a synergistic effect when both the virus receiver and virus spreader wore masks (cotton masks or surgical masks) to prevent the transmission of infective droplets/aerosols (Fig. 2D and E).

We next tested the protective efficacy of masks when the amount of exhaled virus was increased. The viral load was augmented to 10^8^ PFU and exhaled by the spreader; then the uptake of the virus droplets/aerosols was measured when various types of masks were attached to the receiver. As with the lower viral load (5 × 10^5^ PFU) shown in Fig. 2B, the N95 mask sealed with adhesive tape showed approximately 90% protective efficacy (see Fig. 2F and G for a comparison of two N95 products). When the amount of exhaled virus was reduced to 10^5^ PFU or 10^4^ PFU, infectious viruses were not detected, even in the samples from the unmasked receiver (Fig. 2H and I). Viral RNA was detected in all samples; however, due to the quantitative decrease, there was no difference in protective efficacy among all of the masks, including the sealed N95 masks.

Our airborne simulation experiments showed that cotton masks, surgical masks, and N95 masks had a protective effect with respect to the transmission of infective droplets/aerosols and that the protective efficiency was higher when masks were worn by the virus spreader. Considerable viral loads have been detected in the nasal and throat swabs of asymptomatic and minimally symptomatic patients, as well as those of symptomatic patients, which suggests transmission potential (4). Accordingly, it is desirable for individuals to wear masks in public spaces. Importantly, medical masks (surgical masks and even N95 masks) were not able to completely block the transmission of virus droplets/aerosols even when fully sealed under the conditions that we tested. In this study, infectious SARS-CoV-2 was exhaled as droplets/aerosols and mask efficacy was examined. To allow quantification, we conducted our studies by using a relatively high dose of virus, and under these conditions, it is possible that the protective capacity of the masks was exceeded. Although the efficiency of detecting infectious virus was reduced when the amount of exhaled virus was reduced, viral RNA was detected regardless of the type of mask used. These results indicate that it is difficult to completely block this virus even with a properly fitted N95 mask. However, it remains unknown whether the small amount of virus that was able to pass through the N95 masks would result in illness.

It has been reported that the stability of the virus in the air changes depending on the droplet/aerosol components, such as inorganics, proteins, and surfactants, suggesting that the permeation efficiency of masks is also affected by the components of viral droplets/aerosols (5, 6). In our experiments, the virus was suspended in culture supernatant without fetal calf serum or was diluted with phosphate-buffered saline. Further detailed analysis will be required to reveal the precise relationship between the protective efficiency of masks and the components of viral droplets/aerosols.

Our data will help medical workers understand the proper use and performance of masks (e.g., the importance of fitting masks and avoiding their reuse) and to determine whether they need additional protective equipment (e.g., a negative-pressure room or positive-pressure masks) to protect themselves from infected patients.
