# Supplementary material for: Effectiveness of Face Masks in Preventing Airborne Transmission of SARS-CoV-2
Source: mSphere. 2020 Oct 21;5(5):e00637-20. doi: 10.1128/mSphere.00637-20 (PMC7580955; doi:10.1128/mSphere.00637-20)
Supplement: TEXT S1 [file mSphere.00637-20-s0001.docx]

**Materials**

*Cells*

VeroE6/TMPRSS2 (JCRB1819) cells^1^ were obtained from the National Institutes of Biomedical Innovation, Health and Nutrition, Japan. Cells were maintained in Dulbecco’s modified Eagle’s medium (DMEM) containing 10% fetal calf serum (FCS) and antibiotics at 37°C with 5% CO_2_.

*Virus*

The SARS-CoV-2 strain (UT-NCGM02/Human/2020/Tokyo) was isolated from a COVID-19 patient in Japan, and propagated in VeroE6/TMPRSS cells with DMEM and antibiotics as described previously^23^.

*Masks*

The cotton masks were made in the common shape; they comprised a double layer of cotton, with each layer consisting of two layers of 100% cotton gauze, resulting in a total of four layers of gauze, with typical double-strap ties. Surgical masks (typical double-strap tie masks) were purchased from ParaMedical Supplies Corp. The N95 Particulate Respirator and DDO2-N95 masks were purchased from Alpha Pro Tech, Inc. and Shigematsu Works Co., Ltd., respectively.

**Methods**

*A simulation system for airborne transmission of viruses*

For airborne transmission experiments, a test chamber (120-cm long x 40-cm wide x 50-cm high) with an aluminum frame and acrylic panels was constructed in a biosafety cabinet at our biosafety level 3 facility (Figure 1). Proper directional airflow through the safety cabinet containing the test chamber, and the sealability of the test cabinet, were checked by using smoke tests. The relative humidity (RH) and temperature of the test chamber were maintained at 60–65% and 23–25 °C, respectively^4^. Two mannequin heads, which were based on the size and shape of real human heads, were placed facing each other and the distance between them was modifiable (25, 50, or 100 cm).

One mannequin head was connected to a customized compressor nebulizer (NE-C28, Omron) and exhaled a mist of virus suspension through its mouth to mimic a viral spreader. The nebulizer was charged with 6 ml of virus suspension (5 × 10^5^ plaque forming units (PFU) for the experiments described in Figure 2A–E; 1 × 10^8^ PFU for the experiments described in Figure 2F and G; 1 × 10^5^ PFU for the experiments described in Figure 2H; and 1 × 10^4^ PFU for the experiments described in Figure 2I) to generate droplets/aerosols, and exhaled continuously simulating a mild cough at a flow speed of 2 m/s^5^ for 20 minutes. The nebulizer initially sprayed fine droplets/aerosols of virus suspension (mass median diameter, 5.5 ± 0.2 μm; particle sizes, <3 μm: 20%, 3–5 μm: 40%, >5–8 μm: 40%^6^). The particles became smaller as they wafted through the test chamber during experiments.

The other mannequin head, simulating a person exposed to the virus, was connected to an artificial ventilator (SN-480-4, Shinano) through a virus particle-collection unit. Tidal breathing, conducted by the artificial ventilator, was set to a lung ventilation rate representative of a steady state in adults^7^ (i.e., 0.5 L of tidal volume, respiratory rate of 18 breaths/min, and a 50% gas exchange rate). The collection unit employed a gelatin membrane filter (#12602-080-ALK; diameter, 8.0 cm; pore size, 3.0 µm; Sartorius AG) to trap virus particles contained in the inhalation flow. The collection unit was coated with aluminum tape (AL-50BT, 3M) to reduce the static electric charge. The membrane filter to trap the virus particles was immediately dissolved in 10 ml of DMEM containing 5% FCS at 37 °C as described previously^8^, and then the viral loads were assessed by use of a plaque assay to determine the viral titer and a quantitative real-time reverse transcription polymerase chain reaction (qRT-PCR) to determine the copy number of the viral genes.

To test the effectiveness of face masks to prevent the droplet/aerosol transmission of virus, face masks were attached to the mannequin heads according to each manufacturer’s instructions. The N95 mask samples were evaluated under the following two conditions: the masks were naturally fitted along the contours of the mannequin heads, or the edges of the N95 masks were sealed with adhesive tape after placement on the mannequin heads to prevent leakage. To prevent cross-contamination of the infectious virus and viral RNA, the chamber was flushed out with clean air, the inner faces of the chamber and the mannequins were wiped with 80% ethanol and dried with clean air, and all masks were disposed of after each experimental trial.

*Virus titration assay*

Confluent VeroE6/TMPRSS2 cells in 10-cm dishes were infected with 1 ml of a dilution of the dissolved membranes containing viruses. After incubation for 1 h at 37 °C, the virus inoculum was removed and the cells were overlaid with 1% agarose solution in DMEM with 5% FCS. The plates were incubated for 48 h and then the agar-covered monolayers were fixed with 10% neutral buffered formalin. After removal of the agar, the plaques were counted.

*qRT-PCR*

Viral RNA was extracted from 140 μl of the dissolved membranes containing viruses by using the QIAamp Viral RNA Mini Kit (Qiagen). Amplification and detection by qRT-PCR were performed by using the LightCycler 96 System (Roche). qRT-PCR was performed by using the QuantiTect Probe RT-PCR Kit (Qiagen). The probes contained oligonucleotides with the 6-carboxyfluorescein (FAM) reporter dye at the 5′ end and the Black Hole Quencher-1 (BHQ-1) quencher dye at the 3′ end. The primer sequences used were as follows: NIID_2019-nCOV_N forward 5′-AAATTTTGGGGACCAGGAAC-3′; NIID_2019-nCOV_N reverse 5′-TGGCACCTGTGTAGGTCAAC-3′; NIID_2019-nCOV_N probe FAM-ATGTCGCGCATTGGCATGGA-BHQ1-3′. A standard curve was generated from 10-fold serial dilutions of a quantitative synthetic RNA from SARS-related coronavirus 2 (BEI Resources, NR-52358).

*Biosafety statement*

All experiments with SARS-CoV-2 viruses were performed in enhanced biosafety level 3 (BSL3) containment laboratories at the University of Tokyo, which are approved for such use by the Ministry of Agriculture, Forestry, and Fisheries, Japan.

*Statistical analysis*

Data are presented as the mean ± SD for Figure 2A–H. Dunnett's test was performed, and differences were considered to be statistically significant when the *p* value was less than 0.05.

**Supplemental references**

1 Shutoku Matsuyama, Naganori Nao, Kazuya Shirato, Miyuki Kawase, Shinji Saito, Ikuyo Takayama, Noriyo Nagata, Tsuyoshi Sekizuka, Hiroshi Katoh, Fumihiro Kato, Masafumi Sakata, Maino Tahara, Satoshi Kutsuna, Norio Ohmagari, Makoto Kuroda, Tadaki Suzuki, Tsutomu Kageyama, and Makoto Takeda*.* 2020 Mar 31;117(13):7001-7003. *Proc Natl Acad Sci U S A.* Enhanced isolation of SARS-CoV-2 by TMPRSS2-expressing cells. doi:10.1073/pnas.2002589117.

2 Peter J Halfmann, Masato Hatta, Shiho Chiba, Tadashi Maemura, Shufang Fan, Makoto Takeda, Noriko Kinoshita, Shin-Ichiro Hattori, Yuko Sakai-Tagawa, Kiyoko Iwatsuki-Horimoto, Masaki Imai, and Yoshihiro Kawaoka*.* 2020 Aug 6;383(6):592-594. *The New England journal of medicine.* Transmission of SARS-CoV-2 in Domestic Cats. doi:10.1056/NEJMc2013400.

3 Masaki Imai, Kiyoko Iwatsuki-Horimoto, Masato Hatta, Samantha Loeber, Peter J Halfmann, Noriko Nakajima, Tokiko Watanabe, Michiko Ujie, Kenta Takahashi, Mutsumi Ito, Shinya Yamada, Shufang Fan, Shiho Chiba, Makoto Kuroda, Lizheng Guan, Kosuke Takada, Tammy Armbrust, Aaron Balogh, Yuri Furusawa, Moe Okuda, Hiroshi Ueki, Atsuhiro Yasuhara, Yuko Sakai-Tagawa, Tiago J S Lopes, Maki Kiso, Seiya Yamayoshi, Noriko Kinoshita, Norio Ohmagari, Shin-Ichiro Hattori, Makoto Takeda, Hiroaki Mitsuya, Florian Krammer, Tadaki Suzuki, and Yoshihiro Kawaoka*.* 2020 Jul 14;117(28):16587-16595. *Proc Natl Acad Sci U S A.* Syrian hamsters as a small animal model for SARS-CoV-2 infection and countermeasure development. doi:10.1073/pnas.2009799117.

4 Neeltje van Doremalen, Trenton Bushmaker, Dylan H Morris, Myndi G Holbrook, Amandine Gamble, Brandi N Williamson, Azaibi Tamin, Jennifer L Harcourt, Natalie J Thornburg, Susan I Gerber, James O Lloyd-Smith, Emmie de Wit, and Vincent J Munster*.* 2020 Apr 16;382(16):1564-1567. *The New England journal of medicine.* Aerosol and Surface Stability of SARS-CoV-2 as Compared with SARS-CoV-1. doi:10.1056/NEJMc2004973.

5 Hidekazu Nishimura, Soichiro Sakata, and Akikazu Kaga. 2013 Nov 27;8(11):e80244. *PloS one.* A new methodology for studying dynamics of aerosol particles in sneeze and cough using a digital high-vision, high-speed video system and vector analyses. doi:10.1371/journal.pone.0080244.

6 Elna B Berg, and Robert J Picard. J. 2009 Dec;54(12):1671-8. *Respir Care.* In vitro delivery of budesonide from 30 jet nebulizer/compressor combinations using infant and child breathing patterns.

7 Rajeev B Patel, Shaji D Skaria, and Mohamed M Mansour, Gerald C Smaldone. 2016 Jul;13(7):569-76. *J Occup Environ Hyg.* Respiratory source control using a surgical mask: An *in vitro* study. doi:10.1080/15459624.2015.1043050.

8 Etsuko Hatagishi, Michiko Okamoto, Suguru Ohmiya, Hisakazu Yano, Toru Hori, Wakana Saito, Hiroshi Miki, Yasushi Suzuki, Reiko Saito, Taro Yamamoto, Makoto Shoji, Yoshihisa Morisaki, Soichiro Sakata, and Hidekazu Nishimura*.* 2014 Aug 1;9(8):e103560. *PloS one.* Establishment and clinical applications of a portable system for capturing influenza viruses released through coughing. doi:10.1371/journal.pone.0103560.
